# Laser-modified titanium surfaces enhance the osteogenic differentiation of human mesenchymal stem cells

**DOI:** 10.1186/s13287-017-0717-9

**Published:** 2017-11-28

**Authors:** Tatiana A. B. Bressel, Jana Dara Freires de Queiroz, Susana Margarida Gomes Moreira, Jéssyca T. da Fonseca, Edson A. Filho, Antônio Carlos Guastaldi, Silvia Regina Batistuzzo de Medeiros

**Affiliations:** 1 Departamento de Biologia Celular e Genética, CB—UFRN, Universidade Federal do Rio Grande do Norte, Campus Universitário, Lagoa Nova, 59072-970 Natal, RN Brazil; 2 Departamento de Físico-Química, Instituto de Química de Araraquara—UNESP, Araraquara, SP Brazil; 3 Programa de Pós Graduação em Ciências da Saúde, Natal, RN Brazil

**Keywords:** Titanium, Laser beam (Yb-YAG), Surface modification, Human umbilical cord, Mesenchymal stem cells, Osteoinduction, Biocompatibility

## Abstract

**Background:**

Titanium surfaces have been modified by various approaches with the aim of improving the stimulation of osseointegration. Laser beam (Yb-YAG) treatment is a controllable and flexible approach to modifying surfaces. It creates a complex surface topography with micro and nano-scaled patterns, and an oxide layer that can improve the osseointegration of implants, increasing their usefulness as bone implant materials.

**Methods:**

Laser beam irradiation at various fluences (132, 210, or 235 J/cm^2^) was used to treat commercially pure titanium discs to create complex surface topographies. The titanium discs were investigated by scanning electron microscopy, X-ray diffraction, and measurement of contact angles. The surface generated at a fluence of 235 J/cm^2^ was used in the biological assays. The behavior of mesenchymal stem cells from an umbilical cord vein was evaluated using a 3-(4,5-dimethylthiazol-2-yl)-2,5-diphenyltetrazolium bromide (MTT) assay, a mineralization assay, and an alkaline phosphatase activity assay and by carrying out a quantitative real-time polymerase chain reaction for osteogenic markers. CHO-k1 cells were also exposed to titanium discs in the MTT assay.

**Results:**

The best titanium surface was that produced by laser beam irradiation at 235 J/cm^2^ fluence. Cell proliferation analysis revealed that the CHO-k1 and mesenchymal stem cells behaved differently. The laser-processed titanium surface increased the proliferation of CHO-k1 cells, reduced the proliferation of mesenchymal stem cells, upregulated the expression of the osteogenic markers, and enhanced alkaline phosphatase activity.

**Conclusions:**

The laser-treated titanium surface modulated cellular behavior depending on the cell type, and stimulated osteogenic differentiation. This evidence supports the potential use of laser-processed titanium surfaces as bone implant materials, and their use in regenerative medicine could promote better outcomes.

## Background

In recent decades, research into biomaterials has increased, in part to meet demands for materials that will extend the longevity of an ageing population [1]. Concerning the applications of regenerative medicine, a synthetic scaffold should not only be biocompatible and biodegradable to allow native tissue integration, but should also mimic the hierarchical structure of the native tissue. The extracellular matrix is the natural cell scaffold, and it has a wide variety of topographies at the micro/nano scale [2].

Although diverse implantable biomaterials can be used in bone regenerative medicine [3], titanium (Ti) has long been the gold standard for orthopedic and dental approaches [4]. However, several problems related to a loss of aseptic character and implant failure have been described [5]. Furthermore, several critical parameters, such as interactions with body fluids and the physicochemical properties of the implants, are crucial for the longevity and load-bearing capacity of the materials [6]. Cell attachment and cell growth are primarily associated with the chemistry of the material and surface characteristics such as roughness, wettability, and surface energy [7].

Titanium surfaces have been modified by various approaches with the aim of improving the stimulation of osseointegration. Laser beam (Yb-YAG) treatment is a controllable and flexible approach to modifying surfaces, and it can be used in industrial applications [4, 8]. The technique produces a surface with nano-to-micro hybrid structures, high purity, increased surface area, corrosion resistance, biocompatibility owing to the formation of oxide layers, and an increase in bone–implant interactions [8, 9]. The laser irradiation parameters influence surface melting; therefore, it is possible to create different surfaces by simply changing those parameters [8]. It is theoretically possible to develop a surface with characteristics optimized for cell attachment, growth, and/or differentiation.

Human mesenchymal stem cells (hMSCs) have been utilized in numerous studies, including those on bone repair, because they play a crucial role in bone regeneration and fixation [3, 5, 10, 11]. Human bone marrow mesenchymal stem cells (hBM-MSCs) are the most commonly used cells. However, their isolation can be invasive, and their ability to differentiate decreases with age [2, 12, 13]. Neonatal tissues, such as those found in the umbilical cord, are an easily accessible source of hMSCs, and they can be obtained without resorting to painful or invasive techniques. Moreover, they are available in relatively large quantities. It is possible that the hMSCs from umbilical cord tissue are at an earlier stage than cells from adult bone marrow [12]; they therefore have lower immunogenicity, an enhanced proliferation rate, and a greater lifespan [2, 12, 13].

Hybrid hMSC–biomaterial scaffolds therefore have potential for use in bone prosthetics. In situ, cells migrate off the scaffold and undergo differentiation leading to integration of the device and regeneration of the damaged tissue. Furthermore, factors such as the physical properties of the scaffold can stimulate and improve this process [2].

Based on a hybrid hMSC–biomaterial approach, the aim of the present study was to investigate the osteoregenerative effect of a laser-modified nano-to-micro-scale hybrid surface on human umbilical cord mesenchymal stem cells (hUC-MSCs).

## Methods

### Titanium discs

The Ti discs were prepared at UNESP (Araraquara, Brazil). Commercially pure grade II Ti discs (diameter = 15 mm; thickness = 2 mm) were subjected to multipulse Yb:YAG laser irradiation treatment using an OmniMark machine (Omnitek Tecnologia). The Ti discs were polished with abrasive grit (grades 240–600), then treated with laser radiation at various fluences (132, 210, or 235 J/cm^2^). According to the characterization results, the laser-processed titanium (LPT) surface obtained at 235 J/cm^2^ fluence was selected for the biological assays. Untreated Ti discs were used as controls. All of the discs were cleaned and sterilized with gamma radiation.

### Sample characterization

The surface topographies of the Ti discs were investigated by scanning electron microscopy (SEM) (JSM T330A scanning microscope). The crystalline composition of the modified surfaces, such as the types and phases of oxides formed, were analyzed by X-ray diffraction (XRD) using a SIEMENS D5000 X-ray diffractometer (Siemens, Munich, Germany), with angular scanning between 10 and 80°.

The oxide layers were characterized by comparing the obtained data with the standard records in the Committee for Powder Diffraction Studies (CPDS) database. Quantitative phase analysis was carried out using Rietveld refinements [14]. The phases considered are presented in Table 1. The wettability of the samples was evaluated by measuring the contact angle (Ɵ) at room temperature (sessile drop method) using an OCA Contact Angle System (OCA-15 video-based optical contact angle meter). The sessile drop method was applied with ultrapure water and the contact angle was calculated by the Laplace–Young function (SCA 20 software; Dataphysics Instruments GmBh. Germany). The measurement was repeated three times for each sample to obtain the mean value of the contact angle (Ɵ) for the various surfaces (Table 2).

**Table 1:** Crystalline structures of identified phases obtained by laser ablation and percentage of oxide layer in the irradiated titanium surfaces

Phase	Oxide layer (%)		
	132 J/cm^2^	210 J/cm^2^	235 J/cm^2^
α titanium (hexagonal)	47.1	42.2	43.5
β titanium (cubic)	11.2	9.4	5.9
TiO (rhombohedral)	5.3	6.4	11.4
Ti_2_O (rhombohedral)	27.7	38.5	7.3
Ti_3_O (rhombohedral)	–	1.3	30.8
Ti_6_O (rhombohedral)	8.6	2.2	1.0

**Table 2:** Contact angles measured on laser-ablated and titanium control surfaces

Surface	1st measurement	2nd measurement	3rd measurement	Mean ± SD
Titanium control	68.5	65.2	54.8	62.83 ± 7.15
132 J/cm^2^	0	0	0	0
210 J/cm^2^	116.9	111.9	94.5	107.77 ± 11.76
235 J/cm^2^	0	0	0	0

*SD* standard deviation

### Cell culture

Human umbilical cord mesenchymal stem cells (hUC-MSCs) were isolated, characterized, and cultured as described previously [15], and following the Local Ethics Committee directions (FR132464). A Chinese hamster ovary cell line (CHO-k1, ATCC® CCL-61™), kindly provided by Dr Carlos Menck, was cultured as described by de Queiroz et al. [16].

The hUC-MSCs and CHO-k1 cells were seeded onto the Ti discs (10^4^ cells/cm^2^) in complete Dulbecco’s modified Eagle’s medium (DMEM) with high glucose content (DMEM supplemented with 10% fetal bovine serum, 2 mM l-glutamine, 50 U/ml of penicillin, and 50 μg/ml of streptomycin), and grown for 3 h, 1 day, 3 days, and 7 days for adhesion and proliferation analysis by 3-(4,5-dimethylthiazol-2-yl)-2,5-diphenyltetrazolium bromide (MTT) assay (Molecular Probes™), as described previously [16]. Briefly, both cell types were maintained at 37 °C in 5% CO_2_, and the medium was replaced every 3 days. After the exposure times, the medium was removed and a solution of 1 mg/ml MTT was added allowing for 4 h of incubation. The solution was then aspirated and the insoluble formazan crystals were dissolved in 1 ml of DMSO. The optical density was measured at 570 nm. Data were presented as the mean of three independent experiments.

Extracellular mineralization and gene expression were investigated in hUC-MSCs seeded and cultured on the Ti discs for 7 and 14 days in the presence of osteogenic medium (OM). OM comprised complete DMEM supplemented with osteogenic inducers (10^–7^ M dexamethasone, 10 mM glycerophosphate, and 0.2 mM ascorbic acid) (Sigma-Aldrich, St. Louis, MO, USA). We also investigated cells cultured in DMEM without osteogenic inducers as the basal medium (BM).

### Morphology analysis by SEM

The adhesion and morphology of the hUC-MSCs and CHO-K1 cells on the LPT and Ti control surfaces were investigated by SEM after 24 h and 7 days. The samples were fixed with 2.5% glutaraldehyde, treated with 1% osmium tetroxide (OsO_4_) for 30 min, and dehydrated in a series of ethanol solutions (30, 50, 70, 90, and 100%). The samples were visualized using a Quanta 200 SEM (FEI, OR, USA) after gold sputter coating.

### Evaluation of osteogenic differentiation

Alkaline phosphatase (ALP) activity, extracellular matrix mineralization, and the expression of osteogenic gene markers were used to evaluate hUC-MSC differentiation.

#### Alkaline phosphatase activity

ALP activity was measured after 3 and 7 days using an alkaline phosphatase activity kit (Labtest Diagnostica Ltda, Minas Gerais, Brazil), according to the manufacturer’s instructions. Briefly, cells were incubated with 50 μl of substrate and 500 μl of buffer for 30 min. After this period, 1.5 ml of color reagent was added and the ALP activity was measured at 590 nm. The plate culture wells were then washed out with cold PBS and 500 μl of Tris–HCl buffer was added in order to lyse cells and to determine the protein content, using a BCA kit (Bioagency Biotecnologia, São Paulo, Brazil). The measurement was repeated twice with technical triplicate to obtain the mean value and the standard deviation (SD).

#### Extracellular matrix mineralization

The cells were fixed with 70% cold ethanol for 1 h, washed three times with distilled water, and stained with Alizarin Red S (40 mM, pH 4.1) at room temperature for 5 min. The quantitative analysis was carried out as described by Jääger et al. [17]. This analysis was repeated three times.

#### Evaluation of gene expression by quantitative real-time PCR

Total RNA was extracted with a PureLink® RNA mini kit (Thermo Fisher Scientific) and reverse-transcribed using a High Capacity cDNA Reverse Transcription Kit (Qiagen) following the manufacturer’s protocol. Three RNA samples were prepared for each test condition and repeated twice in an independent way. Real-time PCR was performed on a 7500 Fast Real-Time PCR system (Applied Biosystems). The samples were subjected to quantitative real-time polymerase chain reaction (qRT-PCR) using a panel of human osteogenic primers (Table 3). Differences in gene expression on the LPT were evaluated by the ΔΔCt method normalized to glyceraldehyde-3-phosphate dehydrogenase (GAPDH) expression, and reported as the fold change in relation to the Ti controls.

**Table 3:** Human gene primer sequences

Gene	Forward primer (5′–3′)	Reverse primer (5′–3′)
*GAPDH*	AGGTGCGTGTGAACGGATTTG	TGTAGACCATGTAGTTGAGGTCA
*RUNX2*	TCAACGATCTGAAGATTTGTGGG	GGGGAGGATTTGTGAAGACGG
*BMP2*	TTCGGCCTGAAACAGAGACC	CCTGAGTGCCTGCGATACAG
*ALPL*	ACTGGTACTCAGACAACGAGAT	ACGTCAATGTCCCTGATGTTATG
*OCN*	GGCGCTACCTGTATCAATGG	GTGGTCAGCCAACTGGTCA
*OPN*	GAAGTTTCGCAGACCTGACAT	GTATGCACCATTCAACTCCTCG

*GAPDH* glyceraldehyde-3-phosphate dehydrogenase, *RUNX2* runt-related transcription factor 2, *BMP2* bone morphogenetic protein 2, *ALPL* alkaline phosphatase, *OCN* osteocalcin, *OPN* osteopontin

### Statistical analysis

All tests were performed in at least two independent experiments with three technical replicates. The data were analyzed using one-way analysis of variance (ANOVA) (*p* < 0.05) and Tukey’s test for multiple comparisons among groups. Data were expressed as the mean ± SD.

## Results

### Sample characterization

**Fig. 1:**
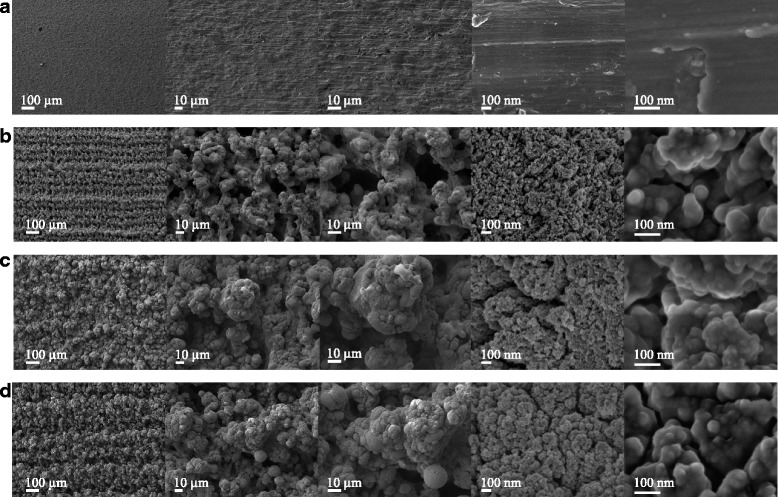
Scanning electron microscopy images of Ti control (**a**), laser-processed titanium (LPT) produced using laser radiation at 132 J/cm^2^ fluence (**b**), LPT produced using laser radiation at 210 J/cm^2^ fluence (**c**), and LPT produced using laser radiation at 235 J/cm^2^ fluence (**d**). Surfaces at × 100, ×500, ×1000, ×50,000, and × 200,000 magnification

**Fig. 2:**
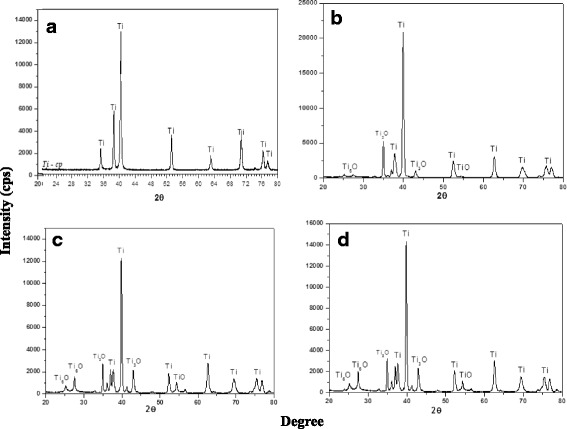
X-ray diffraction spectra of Ti control (**a**), laser-processed titanium (LPT) produced using laser radiation at 132 J/cm^2^ fluence (**b**), LPT produced using laser radiation at 210 J/cm^2^ fluence (**c**), and LPT produced using laser radiation at 235 J/cm^2^ fluence (**d**). cps counts per second, Ti titanium

The laser-treated Ti discs had a complex micro and nano-scaled topography with a typical porous structure and spherical particles (Fig. 1). The crystalline structure confirmed the formation of stoichiometric and nonstoichiometric oxides (Fig. 2). We observed the highest percentage of oxide formation (50.5%) and complete wettability (Ɵ = 0) on the LPT produced by irradiation at 235 J/cm^2^ fluence (Table 1). Therefore, we selected that material for the subsequent evaluation of cellular behavior.

### Cell morphology and proliferation

**Fig. 3:**
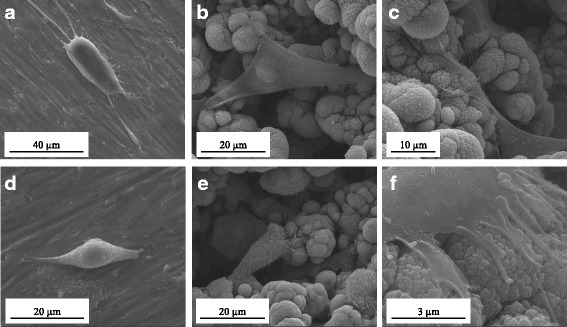
Scanning electron microscopy micrographs of hUC-MSCs cultured after 24 h on Ti control (**a**) and laser-processed titanium (LPT) produced using laser radiation at 235 J/cm^2^ fluence (**b**, **c**); surfaces at × 3000, ×5000, and × 7000 magnification. CHO-k1 cells after 24 h of culture on Ti control (**d**) and LPT produced using laser radiation at 235 J/cm^2^ fluence (**e**, **f**). Surfaces at × 6000, ×5000, and × 40,000 magnification

**Fig. 4:**
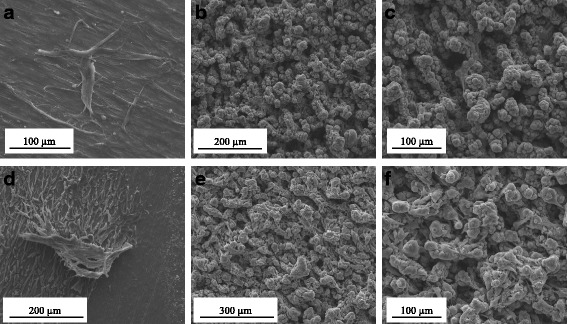
Scanning electron microscopy micrographs after culture for 7 days. hUC-MSCs on Ti control (**a**) and laser-processed titanium (LPT) (**b**, **c**) surfaces. CHO-k1 cells on Ti control (**d**) and LPT (**e**, **f**) surfaces. **a** x1000, **b** x500, **c** x800, **d** x600, **e** x400, **f** x800

SEM revealed morphological differences in the cells after 1 day of growth on the Ti discs (Fig. 3). The CHO-K1 cells and hUC-MSCs cultured on the LPT surface were located mainly in the pores and gaps between the Ti particles (Fig. 3b, e). The cells were well spread, and displayed numerous filopodia (Fig. 3c, f). In contrast, the cells on the Ti controls were round (Fig. 3a, d). After 7 days, the Ti controls were uniformly covered with either type of cells (Fig. 4a, d), whereas cell behavior seemed to depend on cell lineage on the LPT surface (Fig. 4b, e).

**Fig. 5:**
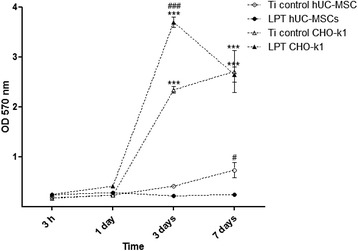
MTT cell metabolic activity assay. hUC-MSC and CHO-k1 cell adhesion and proliferation results on the two different types of titanium discs (laser-processed titanium (LPT) and Ti control) at different times (3 h, and 1, 3, and 7 days). ***Statistically significant differences between cell types at *p* < 0.001; ### represents statistically significant differences between Ti surfaces *p* < 0.001. Data represent means of three independent experiments and SD. MTT, 3-(4,5-dimethylthiazol-2-yl)-2,5-diphenyltetrazolium bromide, OD optical density

The analysis of cell proliferation also revealed a difference between the behaviors of the CHO-k1 cells and the hUC-MSCs (Fig. 5). The LPT surface seems to have improved the proliferation of CHO-k1 cells. The peak of proliferation occurred after 3 days (optical density (OD) = 3.700 for LPT versus OD = 2.345 for the Ti control, *p* < 0.001). At 7 days, the rate of proliferation of CHO-k1 cells decreased to levels similar to those found on the Ti control. SEM analysis revealed no alteration in cell growth between the LPT and the Ti control surface (Fig. 4d, e). On both Ti surfaces, numerous cells were observed distributed uniformly on the surface.

The hUC-MSCs had a slower proliferation rate, and the Ti control surface produced the best results after 7 days (OD = 0.243 for LPT versus OD = 0.733 for the Ti control, *p* < 0.05). SEM analysis revealed cells showed the same behavior observed at MTT assay (Fig. 4a–c). The Ti control surface was uniformly covered and cells reached confluence, while the LPT surface cells did not reach confluence.

### LPT induced osteogenic differentiation in the hUC-MSCs

**Fig. 6:**
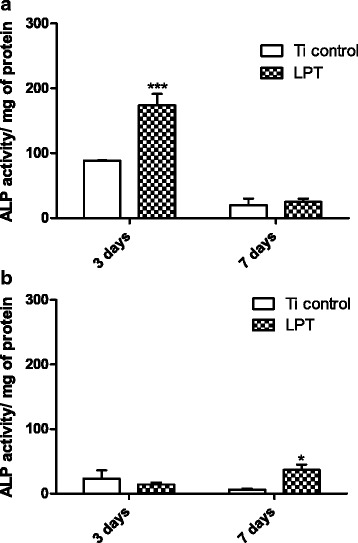
Alkaline phosphatase (ALP) activity. ALP activity in hUC-MSCs after culture in Dulbecco's modified Eagle’s medium on Ti control and laser-processed titanium (LPT) surfaces after 3 and 7 days (**a**); culture of hUC-MSCs in osteogenic medium for the same times on Ti control and LPT surfaces (**b**). Statistically significant differences between Ti surfaces at: ***p < 0.001, *p < 0.05. Values are mean ± SD of two independent experiments. Ti titanium

**Fig. 7:**
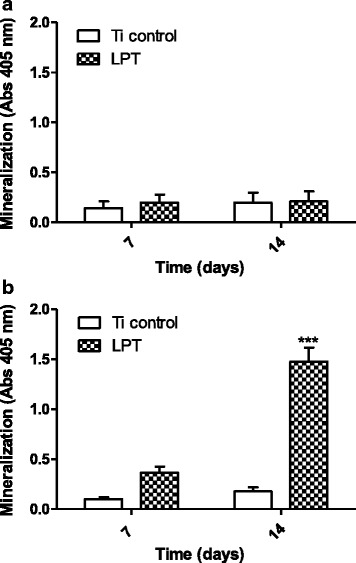
Calcium deposition assay. Alizarin Red S staining of hUC-MSCs on Ti control and laser-processed titanium (LPT) after 7 and 14 days of culture in (**a**) basal medium and (**b**) osteogenic medium.***Statistically significant differences between Ti surfaces at *p* < 0.001. *N* = 3 ± SD. Ti titanium

ALP activity increased in the hUC-MSCs cultured on the LPT in BM. A peak in activity was observed after 3 days (OD = 174.01 ± 17.45 for LPT versus OD = 88.67 ± 0.464 for the Ti control, *p* < 0.01) (Fig. 6a). Curiously, the peak in ALP activity occurred at a later time (i.e., after 7 days) when the cells were maintained in medium with osteogenic inducers (Fig. 6b). Furthermore, the LPT surfaces seem to have promoted the enhancement of extracellular matrix mineralization in the hUC-MSCs after 7 days in BM (OD = 0.1983 ± 0.079 for LPT versus OD = 0.1425 ± 0.069 for the Ti control), and this effect was even more pronounced (i.e., 3.6 times higher) in the presence of osteogenic inducers (OD = 0.3634 ± 0.060 for LPT versus OD = 0.0991 ± 0.020 for the Ti control) (Fig. 7a, b). After 14 days, the difference between the Ti discs was significant (*p* < 0.01) when the cells were incubated in OM (Fig. 7b).

**Fig. 8:**
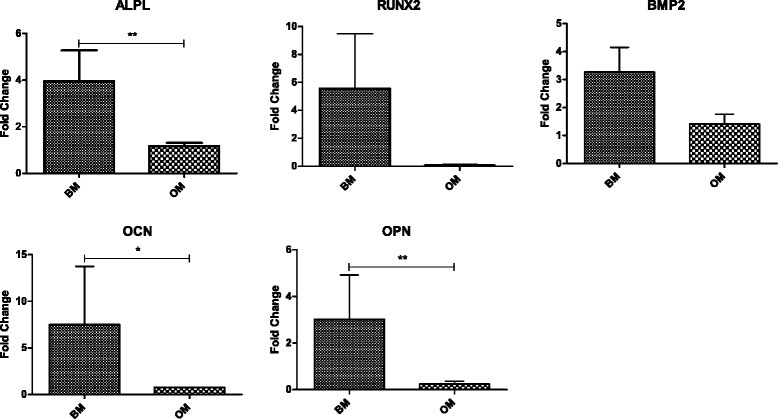
Gene expression of osteogenic markers *ALPL*, *RUNX2*, *BMP2*, *OCN*, and *OPN* in hUC-MSCs following culture on laser-processed titanium (LPT) for 7 days in basal medium (BM) and osteogenic medium (OM). Gene expression evaluated by ΔΔCt method normalized to glyceraldehyde-3-phosphate dehydrogenase (*GAPDH*) expression and reported as fold changes in relation to the Ti control. Statistically significant differences between BM and OM at: **p < 0.01, *p < 0.05. Data presented as mean ± SD (*n* = 2). ALPL alkaline phosphatase, RUNX2 run-related transcription factor 2, BMP2 bone morphogenetic protein 2, OCN osteocalcin, OPN osteopontin

We also investigated osteogenic differentiation by analyzing gene expression to verify the osteoinductive properties of LPT. The canonical osteogenic markers alkaline phosphatase (ALPL), run-related transcription factor 2 (RUNX2), bone morphogenetic protein 2 (BMP2), osteocalcin (OCN), and osteopontin (OPN) were examined over time (7 and 14 days), using *GAPDH* as a housekeeping gene. Gene expression fold change, reported in this work, using Ti control as negative control, showed an increase expression of *ALPL*, *RUNX2*, *OCN*, *BMP2*, and *OPN* in LPT after 7 days in BM. However, in the presence of OM this increase was not observed. In fact, significant differences in the fold changes were observed when cells cultured in BM were compared with those on OM for *ALPL*, *OCN*, and *OPN* (*p* < 0.05) (Fig. 8). No differences in the osteogenic markers expression were observed between cells cultured in BM and OM after 14 days of culture (data not shown).

## Discussion

In recent years, many studies have investigated the influence of the physical and chemical surface characteristics of materials on the biological cascade of events leading to the osteointegration of implants. Many authors agree that the biocompatibility of titanium (Ti) implants depends on the properties of the oxide layer on the surface. Lavisse et al. [18] studied the formation of oxide layers on Ti during laser ablation, and demonstrated the formation of Ti_6_O, Ti_2_O, and Ti_3_O. Several studies have also shown that the formation of an oxide layer improves cell growth on the surface of Ti [9, 11, 19–21], which makes micro-texturing by laser beam (Yb-YAG) an excellent technique for bone medicine.

In the present study, we confirmed the formation of an oxide layer on LPT surfaces (Table 1). Titanium and oxygen were the most common elements found (Fig. 2). No other elements were found on the laser-treated surfaces, revealing a high degree of purity, and showing this process to be without contamination. In agreement with a study by Braga et al. [22], our results showed that the oxidation state of the metallic Ti increased as the fluence of the laser radiation increased; there was a higher degree of oxide layer formation on the Ti surface produced at a laser fluence of 235 J/cm^2^. Biomaterial surfaces interact with water, ions, and numerous biomolecules after implantation. These interactions include hydroxylation of the oxide surface, electrical double-layer formation, protein adsorption, and denaturation, determining how cells and tissues respond to the implant [23].

The topographical analysis of the LPT surfaces showed a complex morphology with micro and nano-scaled patterns. As described by Sisti et al. [4], laser-modified Ti surfaces have distinct topographies with a “cauliflower” morphology that provides a larger surface area and enhanced wettability [4]. Several studies have demonstrated the influence of porous surfaces on cell adhesion [23–26], and our results (Fig. 5c) showed that the cells on the laser-treated Ti surfaces developed numerous filopodia. This confirms that cells on porous surfaces can modify their morphology to follow the surface topography of the sample.

Cell attachment and growth are primarily associated with the chemistry of the material and its surface characteristics. Because cell culture media and body fluids are water based, the wettability of the implant affects the attachment of cells to its surface [27]. The results presented in Table 2 show an improvement in hydrophilicity of the two laser-treated surfaces compared with the Ti controls. Balla et al. [7] concluded that cellular attachment will be poor on any hydrophobic surface with a high contact angle; therefore, we did not culture hUC-MSCs on the Ti surfaces that had been treated by laser ablation at 210 J/cm^2^ fluence because they had unsuitable wettability (the contact angle was higher than 90°). Moreover, low contact angles mean high surface energy, which is another factor that can contribute to better cell attachment [28].

Based on the preliminary studies, the parameters chosen to select the surface used in the experiments with hUC-MSCs were the presence of oxides on the Ti surface and the surface energy. These parameters enabled us to determine the most appropriate Ti surface for our in-vitro studies, which was the surface that had undergone laser ablation at 235 J/cm^2^ fluence.

We used hUC-MSCs and CHO-K1 cells to evaluate the in-vitro biocompatibility of the laser-treated Ti (235 J/cm^2^ fluence). As expected, our experimental data indicated that the CHO-k1 cells grew better on the LPT surfaces over 7 days (Fig. 5). In our previous work [16], we showed that CHO-k1 cells adhere more readily to rough Ti surfaces owing to their greater hydrophilicity. We observed different behavior in the hUC-MSCs over 7 days on the LPT surface. The hUC-MSCs had a lower proliferation rate and better results on the Ti control surface (Fig. 5). The cells did not reach confluence and were distributed in multiple layers inside the porous surface (Fig. 4b, c). Some authors have reported reduced proliferation in cells with osteogenic lineage on rough Ti surfaces compared with on smooth surfaces [6, 29–31]. Therefore, the differences observed in the MTT assay for CHO-k1 cells and hUC-MSCs (Fig. 5) could be related to cell-dependent responses to surface modification. The LPT surface could reduce the proliferation of hUC-MSCs and increase osteogenic differentiation.

hUC-MSCs provide a reproducible cell culture model of osteogenesis, and their in-vitro behavior reflects the influence of surface topography in vivo [32]. The oxide layer, for example, may interact well with nano-scaled proteins, and may also induce hUC-MSCs to differentiate into the osteogenic lineage in vivo [19]. Therefore, we investigated ALP activity as an early osteogenic differentiation marker, and matrix mineralization as a late marker, to evaluate the osteogenic potential of the LPT surface (235 J/cm^2^ fluence).

Our data showed that the ALP activity of the hUC-MSCs on the LPT surface was improved in comparison with those on the Ti control and culture plate (data not shown), even without the addition of an osteogenic inducer. The highest ALP activity occurred after 3 days of culture on the LPT surface (Fig. 6a), but the extracellular mineralization values were similar for both surfaces (Fig. 7a). This behavior was also observed by Fadeeva et al. [33]. However, in the presence of osteogenic inducers, we observed the opposite results for ALP activity and extracellular mineralization (Figs. 6b and 7b). Similar findings for ALP activity, with and without osteogenic inducers, were reported by Sisti et al. [4] after 10 days of culture when no differences in ALP activity in osteogenic medium (OM) was found between laser and machined Ti surfaces. Fadeeva et al. [33] also did not observe ALP activity differences between rough and smooth surfaces in OM. After 7 and 14 days, mouse calvarial osteoblasts seeded on Ti discs presented ALP activity enhanced threefold in cells cultured on rough surfaces compared with osteoblasts cultured on smooth surfaces in OM [8].

There is no consensus in the literature over the effect of rough Ti on ALP activity, mainly due to several variables such as cell type, growth time, and growing conditions. A rough surface has been reported to increase in basal medium (BM) [34] or to not affect in OM [35] the activity of ALP. This shows the importance of this kind of research to improve knowledge in this field.

The discrepancies observed in this work can be attributed to a synergic effect between osteogenic inducers and surface topography stimuli that affects the peak of ALP activity and therefore the differentiation process. In BM, the LPT surface was able to initiate osteoinduction per se*,* but it occurred later than in the presence of OM.

We determined the expression levels of five osteogenic markers (*ALPL*, *RUNX2*, *BMP2*, *OCN*, and *OPN*) to evaluate the responses of hUC-MSCs exposed to an LPT surface at the molecular level. *ALPL* and *RUNX2* are commonly expressed in the early stages of osteogenesis [36]. Our results revealed increased expression of these genes at day 7 in BM and decreased expression at day 14. As described by Sisti et al. [4], RUNX2 is essential for osteoblast maturation; it is a key regulator of OCN and ALPL. OCN and OPN are noncollagenous bone proteins, and are involved in matrix mineralization [35]. The phosphorylated glycoprotein OPN is thought to be present in the early stages of osteogenesis, promoting the attachment of osteoblasts to the extracellular matrix, and it is actively involved in the resorption of bone [4, 36–38]. During the remodeling process, osteoblastic bone formation is associated with osteoclastic bone resorption [36]. Therefore, the surface of the implanted material should be conducive to osteoblast and osteoclast activity [39]. In the present study, the expression levels of both *OCN* and *OPN* were upregulated at 7 days, although we observed an *OCN* peak at 14 days. This upregulation at the mRNA level at day 7 in BM could indicate the induction of hUC-MSC differentiation into osteoblasts following laser beam irradiation.

Perrotti et al. [36] and Jiang et al. [40] also observed a gene expression increase in cells growth on Ti rough surfaces in the absence of osteogenic inductors. Titanium treated with acid and hydrogen peroxide (TiAcidHP) showed an increase expression of osteogenic markers when compared with Ti control in BM. However, this difference was not evident in OM [40]. Gardin et al. [37] also showed an increase on expression of osteoblast markers in human adipose-derived stem cells seeded onto Ti rough surfaces in BM. Similar results were obtained when those cells were seeded on tissue culture plates in the presence of OM. Our gene expression data showed an increase of osteogenic markers in cells cultured in BM on LPT, when compared with Ti control; nevertheless, cells cultured in OM on the untreated surfaces also followed the differentiation pathway due to the presence of inductors in the medium, attenuating the effect of surface topology and resulting in a lower value of fold change. Similar behavior was observed by Wang et al. [35] when no differences in gene expression were observed on rough surfaces in OM.

Our results show that hUC-MSCs cultured on laser-irradiated Ti express osteogenic markers and display ALP activity at an early stage. The main finding of the present study is the osteogenic potential of the material surface itself, which mimics the natural environment of the bone–titanium interface in vivo. ALP activity and osteogenic marker expression were promoted earlier on the LPT, even in early-stage hUC-MSCs, and were less closely associated with an osteogenic lineage.

## Conclusions

Taken together, our results suggest that commercially available pure titanium discs which have been irradiated with a laser beam (Yb-YAG) at 235 J/cm^2^ fluence modulate cellular behavior in a manner that is dependent on the cell type. This clean and reproducible process produces a complex surface topography with micro and nano-scaled patterns, and stoichiometric and nonstoichiometric oxides that improve the hydrophilicity of the LPT surface. Despite low hUC-MSC proliferation, the LPT surface seems to stimulate osteogenic differentiation, leading to an increase in mineralization. This translates into better osseointegration, and demonstrates the potential of a hybrid hUC-MSC–LPT for prosthetic bone devices; its use in regenerative medicine could promote better outcomes.

## Abbreviations

ALP: Alkaline phosphatase
BM: Basal medium
BMP2: Bone morphogenetic protein 2
CHO-k1: Chinese hamster ovary cells
CPDS: Committee for Powder Diffraction Studies
DMEM: Dulbecco’s modified Eagle’s medium
FBS: Fetal bovine serum
GAPDH: Glyceraldehyde-3-phosphate dehydrogenase
hBM-MSC: Human bone marrow mesenchymal stem cell
hMSC: Human mesenchymal stem cell
hUC-MSC: Human umbilical cord mesenchymal stem cell
LPT: Laser-processed titanium
MTT: 3-(4,5-Dimethylthiazol-2-yl)-2,5-diphenyltetrazolium bromide
OCN: Osteocalcin
OM: Osteogenic medium
OPN: Osteopontin
qRT-PCR: Quantitative real-time polymerase chain reaction
RUNX2: Run-related transcription factor 2
SEM: Scanning electron microscopy
Ti: Titanium
XRD: X-ray diffraction
